# Reducing the risks of nuclear war—the role of health professionals

**DOI:** 10.1111/mcn.13554

**Published:** 2023-08-23

**Authors:** Kamran Abbasi, Parveen Ali, Virginia Barbour, Kirsten Bibbins‐Domingo, Marcel G. M. Olde Rikkert, Andy Haines, Ira Helfand, Richard Horton, Bob Mash, Arun Mitra, Carlos Monteiro, Elena N. Naumova, Eric J. Rubin, Tilman Ruff, Peush Sahni, James Tumwine, Paul Yonga, Chris Zielinski

**Affiliations:** ^1^ British Medical Journal; ^2^ International Nursing Review; ^3^ Medical Journal of Australia; ^4^ JAMA; ^5^ Dutch Journal of Medicine; ^6^ London School of Hygiene and Tropical Medicine; ^7^ International Physicians for the Prevention of Nuclear War; ^8^ The Lancet; ^9^ African Journal of Primary Health Care & Family Medicine; ^10^ Revista de Saúde Pública; ^11^ Journal of Public Health Policy; ^12^ New England Journal of Medicine; ^13^ National Medical Journal of India; ^14^ African Health Sciences; ^15^ East African Medical Journal; ^16^ World Association of Medical Editors University of Winchester

In January, 2023, the Science and Security Board of the Bulletin of the Atomic Scientists moved the hands of the Doomsday Clock forward to 90 s before midnight, reflecting the growing risk of nuclear war (Science and Security Board, Bulletin of the Atomic Scientists, 2023). In August, 2022, the UN Secretary‐General António Guterres warned that the world is now in “a time of nuclear danger not seen since the height of the Cold War (UN, 2022). The danger has been underlined by growing tensions between many nuclear armed states (Science and Security Board, Bulletin of the Atomic Scientists, 2023; Tollefson, 2023). As editors of health and medical journals worldwide, we call on health professionals to alert the public and our leaders to this major danger to public health and the essential life support systems of the planet—and urge action to prevent it.

Current nuclear arms control and non‐proliferation efforts are inadequate to protect the world's population against the threat of nuclear war by design, error, or miscalculation. The Treaty on the Non‐Proliferation of Nuclear Weapons (NPT) commits each of the 190 participating nations”to pursue negotiations in good faith on effective measures relating to cessation of the nuclear arms race at an early date and to nuclear disarmament, and on a treaty on general and complete disarmament under strict and effective international control” (UN, 2005). Progress has been disappointingly slow and the most recent NPT review conference in 2022 ended without an agreed statement (Mukhatzhanova, 2022). There are many examples of near disasters that have exposed the risks of depending on nuclear deterrence for the indefinite future (Lewis et al., 2014). Modernisation of nuclear arsenals could increase risks: for example, hypersonic missiles decrease the time available to distinguish between an attack and a false alarm, increasing the likelihood of rapid escalation.

Any use of nuclear weapons would be catastrophic for humanity. Even a “limited” nuclear war involving only 250 of the 13 000 nuclear weapons in the world could kill 120 million people outright and cause global climate disruption leading to a nuclear famine, putting 2 billion people at risk (Bivens, 2022; Xia et al., 2022). A large‐scale nuclear war between the USA and Russia could kill 200 million people or more in the near term, and potentially cause a global “nuclear winter” that could kill 5–6 billion people, threatening the survival of humanity (Bivens, 2022; Xia et al., 2022). Once a nuclear weapon is detonated, escalation to all‐out nuclear war could occur rapidly. The prevention of any use of nuclear weapons is therefore an urgent public health priority and fundamental steps must also be taken to address the root cause of the problem—by abolishing nuclear weapons.

The health community has had a crucial role in efforts to reduce the risk of nuclear war and must continue to do so in the future (Helfand et al., 2022). In the 1980s the efforts of health professionals, led by the International Physicians for the Prevention of Nuclear War (IPPNW), helped to end the Cold War arms race by educating policy makers and the public on both sides of the Iron Curtain about the medical consequences of nuclear war. This was recognised when the 1985 Nobel Peace Prize was awarded to the IPPNW (Nobel Prize Outreach AB, 1985). (http://www.ippnw.org).

In 2007, the IPPNW launched the International Campaign to Abolish Nuclear Weapons, which grew into a global civil society campaign with hundreds of partner organisations. A pathway to nuclear abolition was created with the adoption of the Treaty on the Prohibition of Nuclear Weapons in 2017, for which the International Campaign to Abolish Nuclear Weapons was awarded the 2017 Nobel Peace Prize. International medical organisations, including the International Committee of the Red Cross, the IPPNW, the World Medical Association, the World Federation of Public Health Associations, and the International Council of Nurses, had key roles in the process leading up to the negotiations, and in the negotiations themselves, presenting the scientific evidence about the catastrophic health and environmental consequences of nuclear weapons and nuclear war. They continued this important collaboration during the First Meeting of the States Parties to the Treaty on the Prohibition of Nuclear Weapons, which currently has 92 signatories, including 68 member states (UN Office for Disarmament Affairs, 2023).

We now call on health professional associations to inform their members worldwide about the threat to human survival and to join with the IPPNW to support efforts to reduce the near‐term risks of nuclear war, including three immediate steps on the part of nuclear‐armed states and their allies: first, adopt a no first use policy (Center for Arms Control and Non‐Proliferation, 2023); second, take their nuclear weapons off hair‐trigger alert; and, third, urge all states involved in current conflicts to pledge publicly and unequivocally that they will not use nuclear weapons in these conflicts. We further ask them to work for a definitive end to the nuclear threat by supporting the urgent commencement of negotiations among the nuclear‐armed states for a verifiable, timebound agreement to eliminate their nuclear weapons in accordance with commitments in the NPT, opening the way for all nations to join the Treaty on the Prohibition of Nuclear Weapons.

The danger is great and growing. The nuclear armed states must eliminate their nuclear arsenals before they eliminate us. The health community played a decisive part during the Cold War and more recently in the development of the Treaty on the Prohibition of Nuclear Weapons. We must take up this challenge again as an urgent priority, working with renewed energy to reduce the risks of nuclear war and to eliminate nuclear weapons.

This editorial is being published simultaneously in multiple journals. For the full list of journals see: https://www.bmj.com/content/full-list-authors-and-signatories-nuclear-risk-editorial-august-2023

## AUTHOR CONTRIBUTIONS

Ira Helfand and Andy Haines developed the idea of the editorial and led drafting along with Chris Zielinski. All other authors contributed significantly to the editorial content.

## CONFLICTS OF INTEREST STATEMENT

K. B.‐D. is a full‐time employee of the American Medical Association, working as the editor‐in‐chief of JAMA and the JAMA Network. A. H. is principal investigator of the Pathfinder Initiative 2020–2025, co‐investigator of Sustainable Healthy Food Systems research programme 2017–2023, and co‐investigator of Complex Urban Systems for Sustainability and Health 2017–2023, all funded by the Wellcome Trust, with additional funding from the Oak Foundation for the Pathfinder Initiative, and he reports royalties from Cambridge University Press for the coauthored book Planetary Health; consultancy fees paid to his institution from the Wellcome Trust for his role as Senior Advisor on Climate Change and Health in 2021; travel/meeting support from WHO and Human Frontiers Science Program; and he is a member of the Cool Roofs trial steering committee Nouna Research Centre, Burkina Faso/University of Heidelberg, is co‐chair of the International Advisory Committee, NIHR Clean‐Air (Africa) Global Health Research Unit, is a member of the Independent Advisory Group, Collaboration for the Establishment of an African Population Cohort Consortium, and he was co‐chair of the InterAcademy Partnership, Climate Change and Health Working Group 2019–2022 and co‐chair of the Academy of Medical Sciences/Royal Society working group on “A healthy future—tackling climate change mitigation and human health together” 2020–2021 (all unpaid). I. H. reports honoraria for several speaking engagements, all donated to Back from the Brink, the International Physicians for the Prevention of Nuclear War, or Physicians for Social Responsibility; travel/meeting support for Nobel Peace Laureates' Summit, the World Federation of Public Health Associations World Congress, and the UN Human Rights Commission Youth Summit; and he is a member of the steering committee of Back from the Brink and the International Steering Group of the International Campaign to Abolish Nuclear Weapons, a Board member of the International Physicians for the Prevention of Nuclear War and Physicians for Social Responsibility, and a Trustee of the Phillips Exeter Academy (all unpaid). M. G. M. O. R. reports research grants from the Dutch Research Council, NOW (grant number COMPL.21COV.001) and from the Netherlands Organisation for Health Research ZonMw (grant number 09120012010063) and he is Chair of the Dutch guideline committee on cognitive impairments and dementia. T. R. reports a contract with the Institute for Energy and Environmental Research (USA) for papers addressing the health and environmental consequences of nuclear testing in multiple locations, including Australia, French Polynesia, central Pacific, and China; honorarium from The Choisun Ilbo media group in South Korea for a lecture on nuclear weapons in 2022 and for nuclear weapons presentations from Hyogo Medical Practitioners Association (Japan), Peace Boat (Japan) and the University of Sydney; he was an expert witness on radiation and health for Environmental Justice Australia acting for Mine‐Free Glenaladale regarding proposed Fingerboards Mineral Sands Mine to the Victorian Government Fingerboards Inquiry and Advisory Committee; he is a member of RV3 Rotavirus Vaccine Development Scientific Advisory Board, Murdoch Children's Research Institute/Royal Children's Hospital; he is a member of the Committee of International Campaign to Abolish Nuclear Weapons Australia; he is a member of the Internet Peace Prize Award Committee (Sunfull Foundation, South Korea); he was a member of the Victorian International Humanitarian Law Advisory Committee, Australian Red Cross; he is a Board member of the Initiative for Peacebuilding, Faculty of Arts, University of Melbourne; he is an At‐large Board member of the International Physicians for the Prevention of Nuclear War; he was co‐president of the International Physicians for the Prevention of Nuclear War 2012–23; and he is honorary principal fellow, Melbourne School of Population and Global Health, University of Melbourne. P. Y. reports grants from Atea Pharmaceuticals; honoraria for lectures, presentations, and educational events from bioMérieux and Pfizer Pharmaceuticals; fees for participation on an advisory board from Pfizer Pharmaceuticals; and he is a member of the Antimicrobial Stewardship Study Group Executive Committee (2022–2024) and the Clinical Practice Guideline Panel on Vaccinations in Immunocompromised hosts for the European Society of Clinical Microbiology and Infectious Diseases. C. Z. reports consulting fees for his role as senior adviser on the international journals project from the International Physicians for the Prevention of Nuclear War. The remaining authors declare no conflict of interest.

## References

[mcn13554-bib-0001] Bivens, M. Nuclear famine. IPPNW. August, 2022. https://www.ippnw.org/wp-content/uploads/2022/09/ENGLISH-Nuclear-Famine-Report-Final-bleed-marks.pdf (accessed June 1, 2023).

[mcn13554-bib-0002] Center for Arms Control and Non‐Proliferation . No first use: frequently asked questions. 2023. https://armscontrolcenter.org/issues/no-first-use/no-first-use-frequently-asked-questions/ (accessed June 2, 2023).

[mcn13554-bib-0003] Helfand, I. , Lewis, P. , & Haines, A. (2022). Reducing the risks of nuclear war to humanity. The Lancet, 399, 1097–1098. 10.1016/S0140-6736(22)00422-6 35255264

[mcn13554-bib-0004] Lewis, P. , Williams, H. , Pelopidas, B. , & Aghlani, S. Too close for comfort, cases of near nuclear use and options for policy. Chatham House Report. April, 2014). https://www.chathamhouse.org/2014/04/too-close-comfort-cases-near-nuclear-use-and-options-policy (accessed June 1, 2023).

[mcn13554-bib-0005] Mukhatzhanova, G. 10th NPT Review Conference: why it was doomed and how it almost succeeded. Arms Control Association. October, 2022. https://www.armscontrol.org/act/2022-10/features/10th-npt-review-conference-why-doomed-almost-succeeded (accessed June 2, 2023).

[mcn13554-bib-0006] Nobel Prize Outreach AB . International Physicians for the Prevention of Nuclear War—facts. 1985. https://www.nobelprize.org/prizes/peace/1985/physicians/facts/ (accessed June 1, 2023).

[mcn13554-bib-0007] Science and Security Board, Bulletin of the Atomic Scientists . A time of unprecedented danger: it is 90 seconds to midnight. 2023 Doomsday Clock Statement. Jan 24, 2023. https://thebulletin.org/doomsday-clock/current-time/ (accessed June 1, 2023).

[mcn13554-bib-0008] Tollefson, J. (2023). Is nuclear war more likely after russia's suspension of the new START treaty? Nature, 615, 386. 36882544 10.1038/d41586-023-00679-w

[mcn13554-bib-0009] UN . 2005 Review Conference of the Parties to the Treaty on the Non‐Proliferation of Nuclear Weapons (NPT). May 2–27, 2005. https://www.un.org/en/conf/npt/2005/npttreaty.html (accessed June 2, 2023).

[mcn13554-bib-0010] UN . 2022. Future Generations Counting on Our Commitment to Step Back from Abyss, Lift Cloud of Nuclear Annihilation for Good, Secretary‐General Tells Review Conference, Press Release Aug 1, 2022 SG/SM/21394 https://press.un.org/en/2022/sgsm21394.doc.htm (accessed 10 July 2023).

[mcn13554-bib-0011] UN Office for Disarmament Affairs . Treaties Database. Treaty on the Prohibition of Nuclear Weapons, status of the Treaty. 2023. https://treaties.unoda.org/t/tpnw (accessed June 1, 2023).

[mcn13554-bib-0012] Xia, L. , Robock, A. , Scherrer, K. , Harrison, C. S. , Bodirsky, B. L. , Weindl, I. , Jägermeyr, J. , Bardeen, C. G. , Toon, O. B. , & Heneghan, R. (2022). Global food insecurity and famine from reduced crop, marine fishery and livestock production due to climate disruption from nuclear war soot injection. Nature Food, 3, 586–596. 37118594 10.1038/s43016-022-00573-0

